# *Plasmodium falciparum* Kelch 13 mutations and treatment response in patients in Hpa-Pun District, Northern Kayin State, Myanmar

**DOI:** 10.1186/s12936-017-2128-x

**Published:** 2017-11-25

**Authors:** Craig A. Bonnington, Aung Pyae Phyo, Elizabeth A. Ashley, Mallika Imwong, Kanlaya Sriprawat, Daniel M. Parker, Stephane Proux, Nicholas J. White, Francois Nosten

**Affiliations:** 1 Shoklo Malaria Research Unit, Mahidol-Oxford Tropical Medicine Research Unit, Faculty of Tropical Medicine, Mahidol University, Mae Sot, Thailand; 2 Myanmar Oxford Clinical Research Unit, Yangon, Myanmar; 3 Mahidol Oxford Research Unit, Faculty of Tropical Medicine, Mahidol University, Bangkok, Thailand; 4 Department of Molecular Tropical Medicine and Genetics, Faculty of Tropical Medicine, Mahidol University, Bangkok, Thailand; 5 Centre for Tropical Medicine and Global Health, Nuffield Department of Medicine, University of Oxford, Oxford, UK

**Keywords:** *Plasmodium falciparum* malaria, Artemisinin, Parasite clearance, k13 mutation, Drug resistance

## Abstract

**Background:**

Artemisinin resistance, linked to polymorphisms in the Kelch gene on chromosome 13 of *Plasmodium falciparum* (*k13*), has outpaced containment efforts in South East Asia. For national malaria control programmes in the region, it is important to establish a surveillance system which includes monitoring for *k13* polymorphisms associated with the clinical phenotype.

**Methods:**

Between February and December 2013, parasite clearance was assessed in 35 patients with uncomplicated *P. falciparum* treated with artesunate monotherapy followed by 3-day ACT in an isolated area on the Myanmar–Thai border with relatively low artemisinin drug pressure. Molecular testing for *k13* mutations was performed on dry blood spots collected on admission.

**Results:**

The proportion of *k13* mutations in these patients was 41.7%, and only 5 alleles were detected: C580Y, I205T, M476I, R561H, and F446I. Of these, F446I was the most common, and was associated with a longer parasite clearance half-life (median) 4.1 (min–max 2.3–6.7) hours compared to 2.5 (min–max 1.6–8.7) in wildtype (p = 0·01). The prevalence of *k13* mutant parasites was much lower than the proportion of *k13* mutants detected 200 km south in a much less remote setting where the prevalence of *k13* mutants was 84% with 15 distinct alleles in 2013 of which C580Y predominated.

**Conclusions:**

This study provides evidence of artemisinin resistance in a remote part of eastern Myanmar. The prevalence of *k13* mutations as well as allele diversity varies considerably across short distances, presumably because of historical patterns of artemisinin use and population movements.

**Electronic supplementary material:**

The online version of this article (10.1186/s12936-017-2128-x) contains supplementary material, which is available to authorized users.

## Background

Resistance to the artemisinin derivatives in *Plasmodium falciparum*, characterized by delayed parasite clearance in patients treated with artesunate, emerged in Western Cambodia in 2007 [1–3]. This phenotypic trait was also documented in a large study on the Thai–Myanmar border in 2012 and was [1] found to be heritable [4] and associated with strong selective sweeps in the plasmodial genome [5]. In 2014, non-synonymous single nucleotide polymorphisms in the Kelch propeller domain on chromosome 13 (*k13*) were found to be strongly associated with resistance to artemisinin derivatives [6]. The link between certain *k13* mutations and the clinical resistance phenotype (delayed parasite clearance) has been demonstrated by a number of clinical [7–10], in vitro [11] and transfection studies [12]. In the areas where *k13* mutations have been found, the distribution of different alleles has been variable. For example, the C580Y polymorphism is the most common mutation in Cambodia [13, 14], Laos [15, 16] and Vietnam [16], while the F446I mutants are most common on the Myanmar–China border [9, 17, 18]. On the Thailand–Myanmar border near Mae Sot, the E252Q allele was the most abundant before 2008, but it has since been replaced by C580Y, an allele now close to fixation in this area [19]. The C580Y mutant parasites have emerged at least twice at different locations [20, 21]. Isolates from Cambodia and Vietnam appear to have a common ancestor and they differ from resistant parasites in Myanmar [13, 21]. The C580Y allele found on the Myanmar–China border appears to have originated from the Thailand–Myanmar border [17]; indicating that genetic mutations (at least C580Y) associated with artemisinin resistance in *P. falciparum* are not only emerging “de novo” in different locations but also spreading contiguously.

In 2011, the World Health Organization (WHO) proposed a strategy of containment of artemisinin resistant malaria in the region [22], but this has not contained the spread of resistance; parasites carrying *k13* mutations have now spread to the China–Myanmar [23] and India–Myanmar borders [7, 21, 23, 24]. The C580Y genotype has increased in frequency replacing other genotypes in most of Southeast Asia, and a single C580Y parasite lineage has swept across the Eastern Mekong subregion spreading from Western Cambodia to Southern Vietnam indicating that a “hard” selective sweep has now replaced initial “soft” selective sweeps [19].

More importantly, *k13* alleles under strong selective pressure confer (or are associated with) slow parasite clearance after artemisinin treatment leaving a larger number of surviving (artemisinin resistant) parasites exposed to the partner drugs [25]. The loss in efficacy of DHA-piperaquine in Cambodia and Viet Nam [26, 27] and mefloquine-artesunate on the Thai–Myanmar border [28] have demonstrated how slow parasite clearance due to artemisinin resistance unavoidably leads to the failure of ACT.

**Fig. 1:**
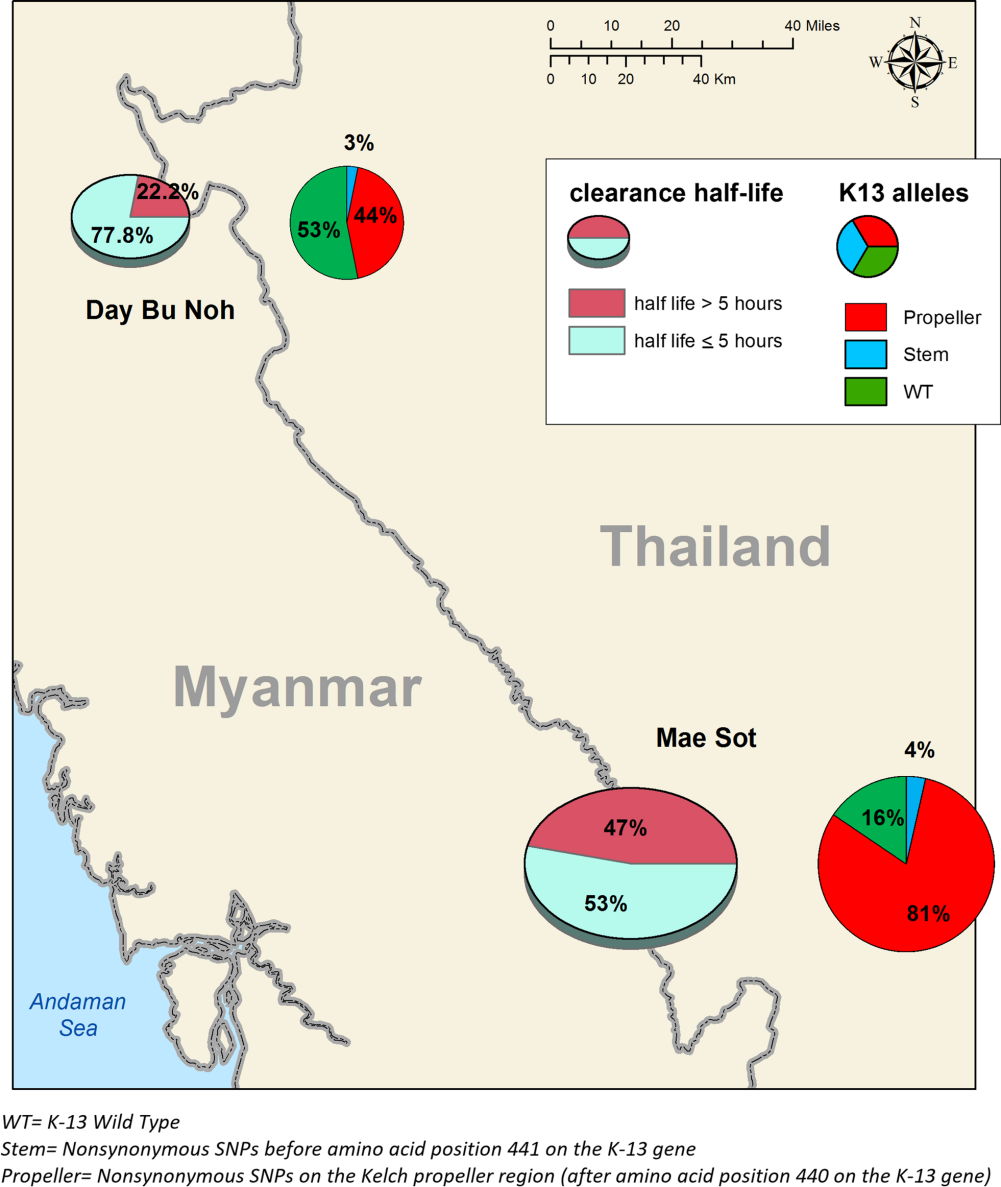
Map showing study site with summary of K-13 mutation and parasite clearance comparison with Maesot

This report presents data collected in 2013 from a remote region along the Myanmar–Thai border in Hpa-pun District, Myanmar (Fig. 1). While artemisinin resistance was well established by 2010 [4] in the border district of Mae Sot (Thailand), 200 km further south, which is a well-connected area encompassing the Trans-Asia highway, data from elsewhere in the remote, hilly and forested parts of eastern Myanmar are few [10, 24]. This study provided an opportunity for examining the phenotype and genetic diversity of *k13* of parasites from a secluded relatively inaccessible region with limited access to ACT (Fig. 1). Parasite clearance half-life and *k13* genotyping results from 35 patients treated with 3 days artesunate monotherapy followed by a full course of ACT are presented.

## Methods

### Study site and study design

This was an open label non-randomized trial designed to evaluate the association between the efficacy of artemisinin against *P. falciparum* malaria (i.e. parasite clearance) and *k13* mutations using the same protocol as a larger multicentre trial [7]. The study was conducted in a remote and isolated region of Hpa-Pun district in Eastern Myanmar in northern Kayin state (Fig. 1). Much of Kayin State has been affected by political instability and conflict for over half a century. The public health infrastructure in this remote district has lagged behind that of other neighbouring areas that have benefited from a relatively more stable situation in recent years. There has been less intense artemisinin usage in the district and malaria remains endemic.

### Patient recruitment

Patients who presented at Day Bu Noh clinic with fever (axillary temperature ≥ 37.5 **°**C) or history of fever within the last 24 h and who were between the ages of 6 months to 65 years were screened for malaria parasites using microscopy of thick and thin Giemsa-stained blood smears. Patients with uncomplicated *P. falciparum* malaria and asexual parasitaemia above or equal to 10,000/µL were enrolled. Pregnant women and patients with severe malaria [29] or treated with an ACT in the past 7 days were excluded. On admission, capillary blood was collected on Whatman^®^ 903 (W-903) filter paper for parasite genotyping. Haemoglobin colour scale Copack^®^ was used for haemoglobin estimation. A clinical examination was also performed, and vital signs were recorded as well as demographic data.

### Drug administration and follow up

All patients received oral artesunate (Guilin Pharmaceutical Co, PRC), 4 mg/kg/day for 3 days. After 3 days of artesunate monotherapy, either

- artesunate-mefloquine (Eloquine^®^ Medochemie Ltd., Cyprus) (4 mg/kg artesunate day 3–5 and mefloquine 15 mg/kg day 4 & 10 mg/kg day 5) or
- dihydroartemisinin-piperaquine (Duo-Cotecxin^®^ Beijing Holley-Cotec Pharmaceuticals Co., Ltd, China) at a dose of 2.5 mg/kg dihydroartemisinin and 20 mg/kg piperaquine day 3–5 was administered.

All treatments were supervised. Peripheral parasitaemia and temperature were measured at 0, 4, 6, 8, 12 and every 6 h thereafter until two consecutive negative slides were obtained. No further follow up was undertaken after the patient finished the full course of treatment and had a negative malaria slide on two occasions. Other clinical data and vital signs were recorded daily.

### Sequencing of the *Plasmodium falciparum kelch13* gene

Kelch genotyping was performed at the malaria molecular laboratory of Mahidol Oxford Tropical Medicine Research Unit using the method reported previously [24, 28] (see more detail in Additional file 1).

### Data management and statistical analysis

Patient data were recorded on individual Case Report Forms (CRFs) and later entered into a Microsoft Access database and analysed using STATA (Version 13, STATA Corp) and Graphpad prism (version 5). Normally distributed data were compared by Student’s t test and non-normally distributed data by the Wilcoxon rank-sum (Mann–Whitney) test. Parasite clearance half-lives were calculated using the World Wide Antimalarial Resistance Network (WWARN) Parasite Clearance Estimator [2, 30].

### Ethical approval

Written informed consent was obtained after the screening process and the study was explained in detail through the use of a local interpreter. Consent was obtained from a parent or guardian for participants below 18 years.

Ethical approval was given by the Oxford Tropical Research Ethics Committee (OXTREC 06-11). Verbal approval was given locally by existing health authorities KDHW (Karen Department of Health and Welfare) in the absence of a local ethics committee. However, a community advisory board composed of members of the local population also approved this study [9].

## Results

### Patients disposition

**Fig. 2:**
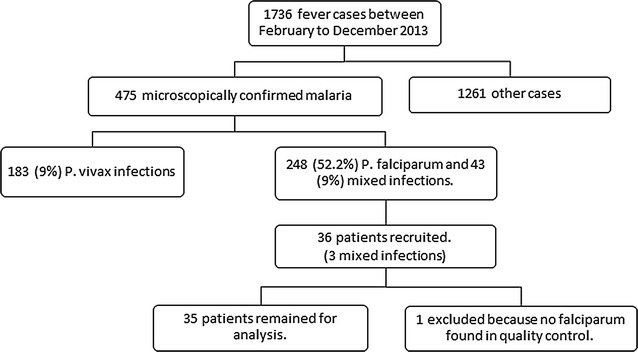
Study flow diagram

Between February and December 2013, a total of 1736 febrile patient presented at the clinic, and 27.4% (475) of them were microscopically confirmed to have malaria (Fig. 2). Of the 291 patients with *P. falciparum* malaria, 36 that met the inclusion criteria and gave consent were recruited into the study (including 3 mixed infections). The main reason for exclusion was low parasitaemia. The baseline demographic and clinical data of the patients are summarized in Table 1.

**Table 1:** Baseline demographic and clinical data of recruited patients

Screened, n	291
Recruited, n	35
Male, n (%)	20 (57.1)
Age, years	
Median (IQR)	14.0 (8.5)
< 5, n (%)	2 (5.6)
5–15, n (%)	17 (47.2)
> 15, n (%)	17 (47.2)
Weight, kg	
Median (IQR)	41.0 (28.0)
Clinical profiles (Day 0)	
Proportion of patients with fever	
n (%)	23 (65.7%)
Temperature	
Mean (SD)	38.5 (0.8)
Haemoglobin (g/dL)	
Median (IQR)	12.0 (0.5)
Parasite density	
Geometric mean (per µL)	86,063
Min	22,608
Max	310,860
Gametocytaemia	
n (%)	3 (8.3%)

All patients had a history of fever before coming to the clinic and 23/35 (65.7%) had fever on admission. By 48 h 30/35 (85.7%: 95% CI 74.1–97.3) of the patients were afebrile and all had cleared their fever by 72 h. After 3 days of artesunate, 13 (37.1%; 95% CI 21.1–53.1%) patients remained parasitaemic but all were negative by microscopy by day 4. Three (8.6%) patients had gametocytes on admission that were cleared in 2 patients by day 6.

### Prevalence of *k13* mutations

**Fig. 3:**
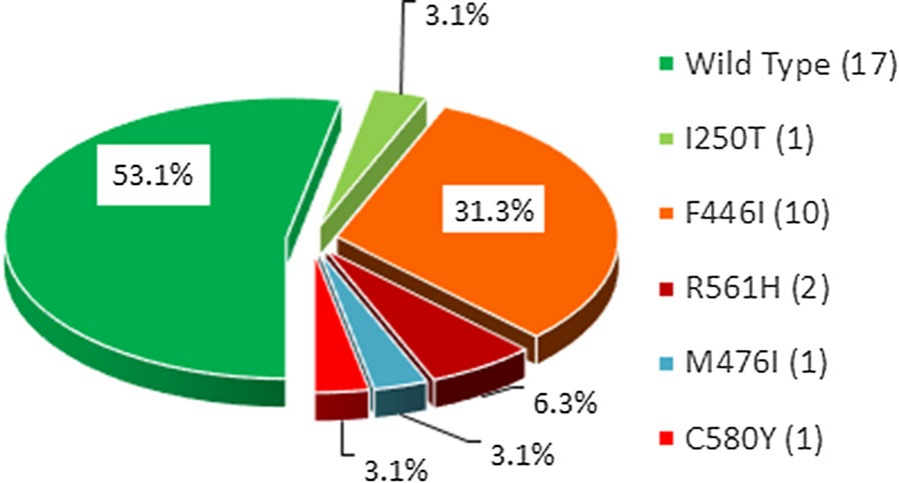
Proportion of K-13 SNPs in recruited patients

From the 35 recruited patients, 32 (91.4%) parasite isolates were successfully genotyped for *k13* sequence. Five different non-synonymous polymorphisms were detected in 46.9% (15/32; 95% CI 29.6–64.2%) of samples including 4 in the propeller region and 1 in the ‘stem’ region of the kelch protein. No sample had more than one mutation. Among these, the most common allele was F446I (31.3%) (Fig. 3).

### Association of *k13* genotype and treatment outcomes

**Fig. 4:**
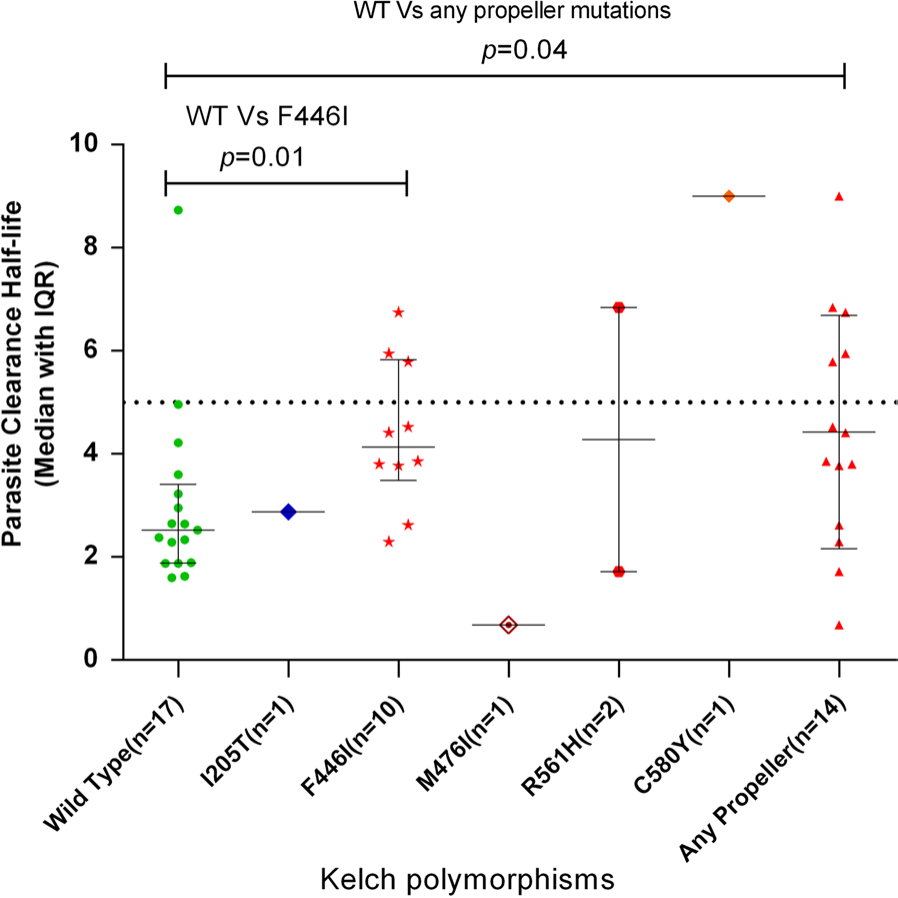
Comparison of parasite clearance half-lives of different K-13 SNP

There was a statistically significant difference in the parasite clearance half-life (median, IQR) of patients infected with wild type *k13* (2.5, 1.3 h) compared to those infected with isolates carrying a mutation in the propeller region (4.1, 3.3 h) (p = 0.04). Again, the patients infected with the most common allele, F446I cleared their parasites more slowly (4.1, 2.0 h) (p = 0.04) compared to those infected with wild type K-13 (Fig. 4; Additional file 2). The longest parasite clearance half-life (9.0 h) was in the patient infected with a parasite carrying the C580Y mutation.

### Safety

The 6-day treatment (3-day artesunate monotherapy follow by 3-day ACT) was well tolerated. None of the patients developed severe malaria. There were no serious adverse events.

## Discussion

Throughout South East Asia parasite clearance rates are decreasing among patients with uncomplicated *P. falciparum* malaria, a pattern that is strongly associated with mutations in the *k13* gene of *P. falciparum* and accompanying partner drug resistance. Until now, 108 non-synonymous polymorphisms of *k13* gene have been found in all malarious regions of the world. Most of the mutations in the propeller region of the gene are associated with the slow parasite clearance phenotype, and in Asia show evidence of recent selection [16, 19]. Historical data from the Thai–Myanmar border (Mae Sot area) show that the prevalence of *k13* mutations has increased significantly, and among them the C580Y allele has out-competed others in recent years signifying that a “hard” selective sweep has replaced an initially “soft” one [19]. The C580Y mutation also dominates in the Eastern Greater Mekong sub-region although this successful lineage had a different genetic origin to the lineage in Eastern Myanmar. In this study in a relatively inaccessible region 200 km away but days travel from Mae Sot, the most frequent kelch mutant allele was F446I. This is the predominant allele in the North of Myanmar which was significantly associated with slow parasite clearance.

The main limitation of this study is the small sample size, which is related to both the remoteness of the study site, logistical difficulties related to working in this remote area, and the relatively high parasitaemia threshold for inclusion (i.e. ≥ 10,000/µL). The parasitaemia threshold was chosen in order to obtain reliable parasite clearance curves.

However, this small study highlights the complexity of the spatial dynamics of artemisinin resistance in *P. falciparum* with significant differences in allele frequencies across small distances. Even though the prevailing alleles may be different it is clear that artemisinin resistance has emerged even in geographically secluded areas and ultimately may be unstoppable. In the absence of alternative treatments ready to replace the failing ACT, elimination remains the best practical strategy to slow down or halt progression of artemisinin resistance in this region.

## Additional files

**Additional file 1.** Genetic markers of artemisinin resistance.

**Additional file 2.** Parasite clearance curves for individual patients.
