# Supplementary material for: Plasmodium falciparum Kelch 13 mutations and treatment response in patients in Hpa-Pun District, Northern Kayin State, Myanmar
Source: Malar J. 2017 Nov 25;16:480. doi: 10.1186/s12936-017-2128-x (PMC5702082; doi:10.1186/s12936-017-2128-x)
Supplement: Supplementary file 1 — Additional file 1. Genetic markers of artemisinin resistance. [file 12936_2017_2128_MOESM1_ESM.docx]

# Additional Methods

## Genetic markers of artemisinin resistance

Kelch genotyping was done at malaria molecular laboratory of Mahidol Oxford Tropical Medicine Research Unit. DNA from admission blood spots was extracted using the QIAamp® DNA Blood Mini Kit (QIAGEN, Germany) following the manufacturer’s instructions. K13 propeller region (1725980-1726940bp, positions 419-707) was amplified by using two primer sets: fragment 1 (1725980-1726520bp, pos 419-570): F- ATCTAGGGGTATTCAAAGG, R- CCAAAAGATTTAAGTGAAAG; fragment 2 (1726400-1726940bp, pos 545-707): F-CTGCCATTCATTTGTATCT, R- GGATATGATGGCTCTTCTA. We also amplified the 5' region: fragment 3 (1725380-1725680bp, pos 211-302) using primers F- TGAAAATATGGTAGGTGATT and R- ATCGTTTCCTATGTTCTTCT. PCR products were treated with Exo-SAP-IT (GE Healthcare), and sequenced directly in both directions using the BigDye Terminator v3.1 cycle sequencing kit (Applied Biosystems, Inc., Foster City, CA). BigDye products were cleaned using the BigDye XTerminator Purification kit (ABI, USA) and then run on a ABI 3730 capillary sequencer. The data were aligned and analysed using SeqScape version 2.7.
