# Supplementary figures and images for: Plasmodium falciparum Kelch 13 mutations and treatment response in patients in Hpa-Pun District, Northern Kayin State, Myanmar
Source: Malar J. 2017 Nov 25;16:480. doi: 10.1186/s12936-017-2128-x (PMC5702082; doi:10.1186/s12936-017-2128-x)

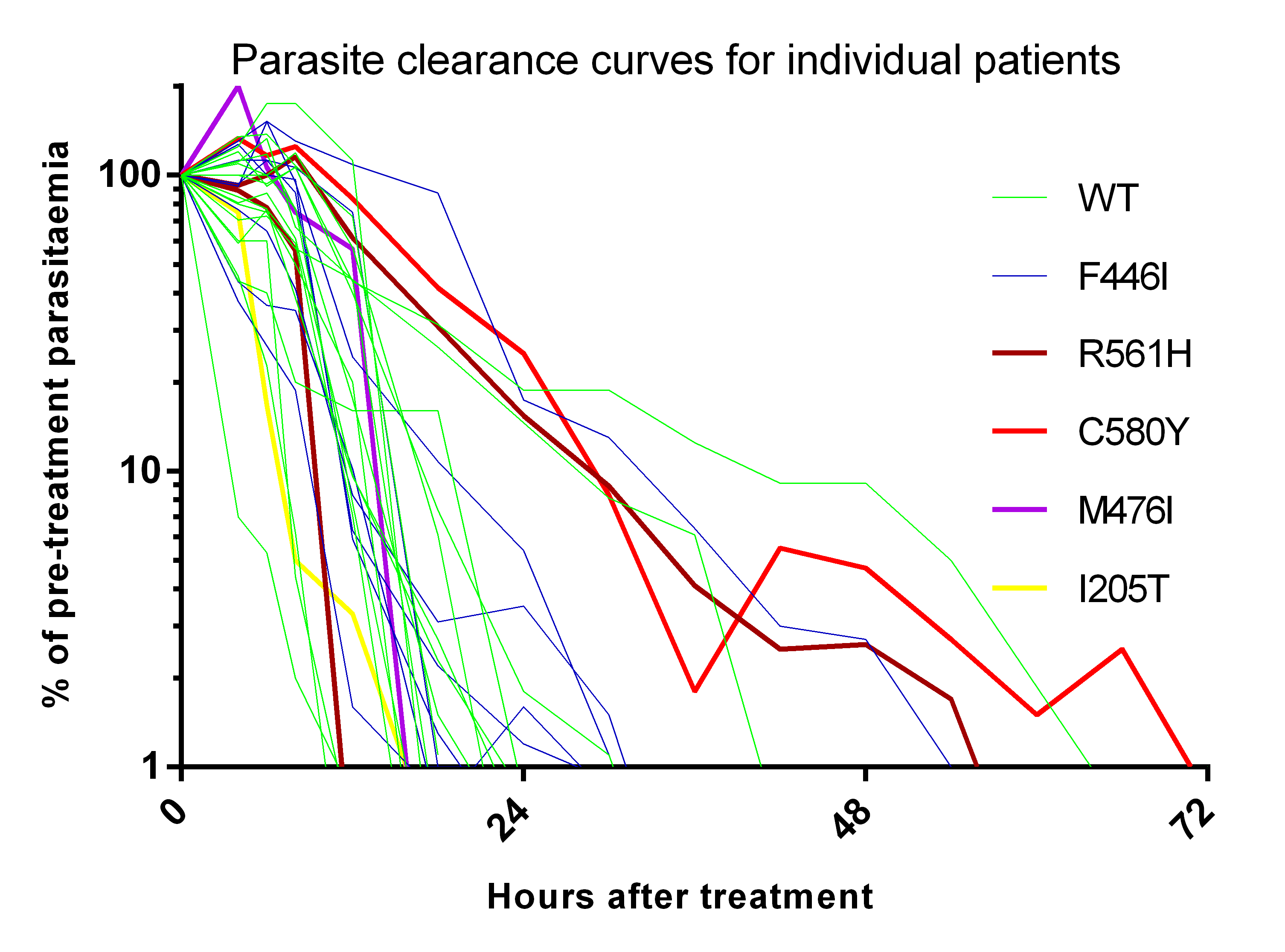

Supplement: Supplementary file 2 — Additional file 2. Parasite clearance curves for individual patients. [file 12936_2017_2128_MOESM2_ESM.png]
